# Fusion of myofibre branches is a physiological feature of healthy human skeletal muscle regeneration

**DOI:** 10.1186/s13395-023-00322-2

**Published:** 2023-08-12

**Authors:** Grith Højfeldt, Trent Sorenson, Alana Gonzales, Michael Kjaer, Jesper L. Andersen, Abigail L. Mackey

**Affiliations:** 1 Department of Orthopaedic Surgery, Institute of Sports Medicine Copenhagen, Copenhagen University Hospital — Bispebjerg and Frederiksberg, Nielsine Nielsens Vej 11, 2400 Copenhagen, NV Denmark; 2 Department of Biomedical Sciences, Faculty of Health and Medical Sciences, University of Copenhagen, Blegdamsvej 3B, 2200 Copenhagen N, Denmark; 3 Department of Clinical Medicine, Faculty of Health and Medical Sciences, University of Copenhagen, Belgdamsvej 9, 2100 Copenhagen Ø, Denmark

**Keywords:** Muscle regeneration, Muscle injury, Electrical stimulation, Muscle fibre branching, Muscle fibre splitting, Muscle fibre typing

## Abstract

**Background:**

The occurrence of hyperplasia, through myofibre splitting, remains a widely debated phenomenon. Structural alterations and fibre typing of skeletal muscle fibres, as seen during regeneration and in certain muscle diseases, can be challenging to interpret. Neuromuscular electrical stimulation can induce myofibre necrosis followed by changes in spatial and temporal cellular processes. Thirty days following electrical stimulation, remnants of regeneration can be seen in the myofibre and its basement membrane as the presence of small myofibres and encroachment of sarcolemma and basement membrane (suggestive of myofibre branching/splitting). The purpose of this study was to investigate myofibre branching and fibre type in a systematic manner in human skeletal muscle undergoing adult regenerative myogenesis.

**Methods:**

Electrical stimulation was used to induce myofibre necrosis to the vastus lateralis muscle of one leg in 5 young healthy males. Muscle tissue samples were collected from the stimulated leg 30 days later and from the control leg for comparison. Biopsies were sectioned and stained for dystrophin and laminin to label the sarcolemma and basement membrane, respectively, as well as ATPase, and antibodies against types I and II myosin, and embryonic and neonatal myosin. Myofibre branches were followed through 22 serial Sects. (264 μm). Single fibres and tissue blocks were examined by confocal and electron microscopy, respectively.

**Results:**

Regular branching of small myofibre segments was observed (median length 144 μm), most of which were observed to fuse further along the parent fibre. Central nuclei were frequently observed at the point of branching/fusion. The branch commonly presented with a more immature profile (nestin + , neonatal myosin + , disorganised myofilaments) than the parent myofibre, together suggesting fusion of the branch, rather than splitting. Of the 210 regenerating muscle fibres evaluated, 99.5% were type II fibres, indicating preferential damage to type II fibres with our protocol. Furthermore, these fibres demonstrated 7 different stages of “fibre-type” profiles.

**Conclusions:**

By studying the regenerating tissue 30 days later with a range of microscopy techniques, we find that so-called myofibre branching or splitting is more likely to be fusion of myotubes and is therefore explained by incomplete regeneration after a necrosis-inducing event.

**Supplementary Information:**

The online version contains supplementary material available at 10.1186/s13395-023-00322-2.

## Background

Work from the late nineteenth century directed the hypothesis that we are born with a set number of myofibres, and that muscle growth is caused by an increased cell (myofibre) mass [1]. However, since then, early human and rodent studies pointed towards an ability for muscle fibres to increase in number immediately post birth [2, 3]. Research in murine Duchenne muscular dystrophy models has reported longitudinal fibre splitting not observed in control mice [4, 5]. Similar observations have been made in muscle samples from two young boys with muscular dystrophy [6]. This has led some to hypothesise that, in the adult state, muscle can undergo not only hypertrophy (increased cell size) but also a form of hyperplasia through muscle fibre splitting [7, 8]. While Duchenne represents a pathological condition characterised by chronic cycles of degeneration and regeneration [9], there is support for physiological fibre splitting in animals subjected to extreme loads, such as in rodents [10, 11], birds [12, 13] and amphibians [14]. Just as fibre hyperplasia is a common developmental feature of drosophila [15].

Rodent and human studies generally agree that the processes involved in muscle injury, repair and regeneration are conserved across species [16], with most of the variation being accounted for by differences in the models employed. However, with regard to the question of hyperplasia vs. hypertrophy, data in humans are less clear than in animals, which limits the translation of concepts of muscle growth between species. In human powerlifters, indirect measures suggest the occurrence of hyperplasia. For example, in individuals who have performed heavy strength training for years, microscopy analysis of muscle tissue sections clearly shows visible clefts formed by encroachment of the myofibre membrane into the myofibre [17]. In addition, powerlifters have larger total muscle size compared to previously untrained individuals who completed 6 months of resistance training, despite similar muscle fibre size between the two groups [18]. Thus, either the powerlifters are born with more fibres or they create more. It should be noted, however, that some of the powerlifters were current or previous users of anabolic steroids and showed a higher number of central nuclei than what is normally seen [19, 20], which could be indicative of some degree of pathology, or, as we propose here, regeneration.

Indeed, the appearance of split or branched myofibres has been attributed to incomplete lateral fusion of myotubes during regeneration [21, 22]. This is in line with observations in human vastus lateralis muscle regenerating after myofibre necrosis induced by neuromuscular electrical stimulation [23, 24]. However, this has not been studied in a systematic manner, and as such represents an open question, which we address in this study. Additional unanswered questions relate to whether type II muscle fibres are more susceptible to injury and how to evaluate fibre type reliably during the highly dynamic process of muscle fibre regeneration, from expression of developmental myosins to their replacement by mature myosins organised in strict sarcomere register. The purpose of this study was therefore to investigate myofibre branching and fibre type in a systematic manner in human skeletal muscle undergoing adult regenerative myogenesis.

## Methods

### Subjects and experimental design

The study was approved by the Regional Scientific Ethical Committees of Copenhagen in Denmark (Ref: HD-2008–074) and conducted in accordance with the Declaration of Helsinki. The muscle biopsies analysed in this study are a subset of samples collected during a larger study [25]. Briefly, volunteers were all young healthy males subjected to a muscle injury protocol consisting of 200 electrically stimulated eccentric contractions of the vastus lateralis muscle of one leg, as described in detail [25].

For the present study, 5 subjects were selected based on the availability of muscle tissue collected from the injured leg on day 30 and the control leg of the same individual, as well as evidence of necrosis on cross-sections on day 7. The subject characteristics and… are shown in Table 1.

**Table 1:** Subject characteristics and muscle injury markers 4, 7 and 30 days after injury

	Mean		SD		Median		SD
Age [years]	22	±	5	Dystrophin neg. fibres day 7 [%]	12	±	7
BMI [kg/m^2^]	22	±	2	nMHC pos. day 7 [%]	2	±	6
Height [m]	1.78	±	0.06	nMHC pos. day 30 [%]	52	±	26
Weight [kg]	70	±	8	Plasma creatine kinase day 4 [U/l]	27,400	±	9365

Age and anthropometric values are mean ± SD. Injury markers are median ± SD

### Muscle biopsy sampling

Muscle biopsies were collected from the vastus lateralis using the percutaneous needle biopsy technique of Bergström [26]. Local anaesthetic (1% lidocaine: Amgros I/S, Copenhagen, Denmark) was applied subcutaneously, and tissue was extracted with 5–6-mm diameter biopsy needles and manual suction. Biopsy tissue was then divided into portions and preserved appropriately for cryosectioning, single fibres or transmission electron microscopy (TEM). For cryosectioning, tissue was embedded in Tissue-Tek (Sakura Finetek Europe, Zoeterwoude, the Netherlands) and frozen in isopentane, precooled by liquid nitrogen and stored at − 80 °C. For single fibres, fibre fascicles were fixed in Zamboni fixative (2% formaldehyde, 0.15% picric acid) and stored at − 20 °C in 50% glycerol in PBS [24, 27]. For TEM, a small part of the tissue sample was immersed in 2% glutaraldehyde in 0.05-M sodium phosphate buffer (pH 7.2) and stored at 4 °C.

### Cryosectioning

A 12-μm-thick sections were cut from frozen samples in a cryostat, placed on glass slides (Superfrost Plus) and stored at − 80 °C. Two serial sections were cut for each slide. In total, twenty-two such slides were prepared from each sample for a series of histochemical (ATPase) and immunofluorescence staining, as indicated in Supplemental Table 1, resulting in 44 serial sections (covering a depth of 528 μm) in total from each specimen.

### Cryosection immunofluorescence

Fourteen slides from each sample were immunofluorescently stained for MyHC I (myosin heavy chain type I), MyHC II (myosin heavy chain type II), MyHCe (myosin heavy chain-embryonic) or MyHCn (myosin heavy chain-neonatal). Along with these target proteins, the sarcolemma and basement membrane were labelled by antibodies against dystrophin and laminin, respectively (Supplemental Table 2). Primary antibodies were applied overnight to fixed (Histofix, 10 min), or unfixed, sections according to the respective antibody use instructions. Sections were then incubated in a cocktail of three secondary antibodies (Supplemental Table 2), for 45 min before being mounting in ProLong Gold Antifade Reagent, containing DAPI (Invitrogen, P36931).

### Cryosection ATPase histochemistry

Eight slides were stained by ATPase histochemistry conducted at pH 9.4 after both alkaline (pH 10.30) and acid (pH 4.37, 4.53 and 4.58) pre-incubation [28, 29].

### Cryosection microscopy

ATPase and immunofluorescent cryosections were viewed on a widefield microscope (Olympus BX51). Brightfield and fluorescent images were captured by an Olympus DP71 camera (Olympus Deutschland GmbH, Hamburg, Germany), controlled by the Olympus cellSens software.

### Cryosection fusion/branching analysis

**Fig. 1:**
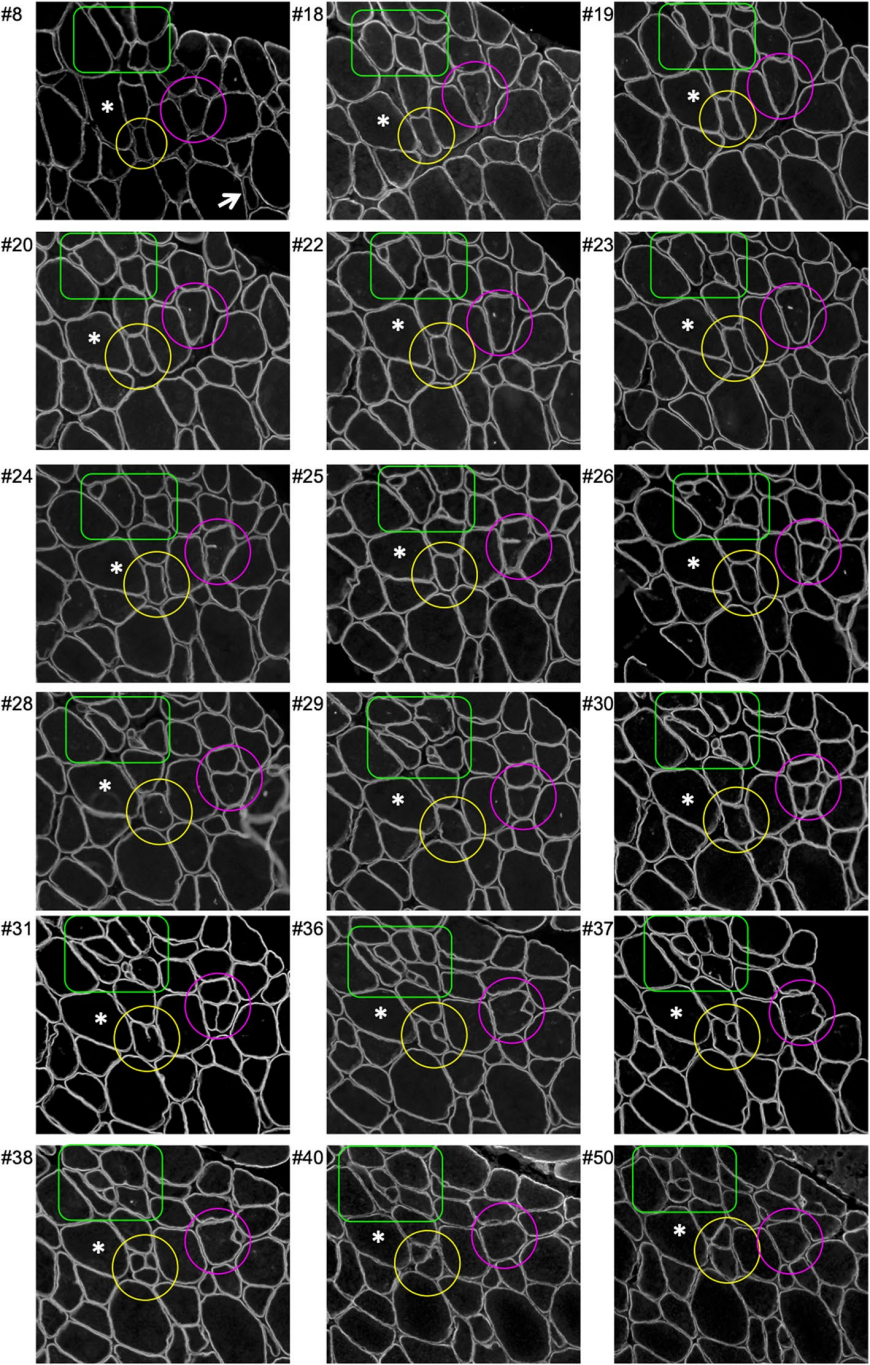
Cross-sectional profiles of regenerating fibres. A 12-μm-thick serial sections of a biopsy from regenerating, healthy, human vastus lateralis muscle 30 days after necrosis induced by electrically stimulated eccentric contractions. Sections were stained with dystrophin to label the sarcolemma. Note the change in myofibre shape and features of branching and fusion in each highlighted area (coloured shape outlines). *Indicates the same (uninjured) myofibre throughout the series for references. Serial section numbers are indicated. Scale bar, 100 μm

Viewing the serial sections of the regenerating samples (Fig. 1), several clear cases of myofibre branching or fusion were apparent, for example an average-sized fibre on one section appearing as several small fibres on subsequent sections. To study this in a systematic manner, such fibres were identified and followed through serial sections for as long as they could be followed or until we reached the last section in the series. Using staining for laminin and dystrophin, patterns of fibre branching or fusion were recorded. The onset of fibre branching was defined as a point where the sarcolemma (dystrophin) formed a cleft in the fibre, as described by Swash and Schwartz in their study of myopathic disorders [30]. These clefts often developed further on subsequent sections and eventually extended fully through the fibre, resulting in what appeared to be two separate and fully dystrophin-enclosed fibres [30]. In recording branching/fusing events, the fibre branch length was calculated by locating the first section showing a dystrophin cleft. The termination of the branch was recorded as the section where the branch appeared to have fused with the parent fibre (no dystrophin cleft). Some branches remained as branches in the last available section, so their measured length was classified as a minimum length, whereas some branches could not be followed confidently (for example due to folds in the section) and were abandoned. In the end, twelve fibres were identified on sections from two participants and followed through 22 serial sections, covering 264 μm of tissue depth (starting with section no. 13, according to the overview presented in Supplemental Table 1). To view such branches longitudinally, we next examined the single fibres.

### Single fibre immunofluorescence and microscopy

Single muscle fibres were isolated from one participant and stained as described [24, 31]. Briefly, fibres were incubated overnight in primary antibodies, 2 h in secondary antibodies (with or without phalloidin; Supplemental Table 3) and 5 min in Hoechst 33,342 (1 μg/ml, H1399; Invitrogen A/S, Taastrup, Denmark). The fibres were mounted (Prolong Gold mounting medium, Molecular Probes, P36930) on a microscope slide and stored at − 20 °C. Confocal images of single fibres were acquired with a Zeiss LSM710, with objectives as follows: 20 × /0.8 Plan-Apochromat, 40 × /1.3 oil DIC EC Plan-Neofluar, 63 × /1.4 oil DIC Plan-Apochromat. Hoechst, Alexa Fluor 488 and Alexa Fluor 568 were excited by the lasers 405 nm Diode, 488 nm argon and 543 nm HeNe, respectively.

### Transmission electron microscopy and tissue processing

After 3 rinses in 0.15-M sodium phosphate buffer (pH 7.2), the tissue samples were postfixed in 1% OsO_4_ in 0.15-M sodium phosphate buffer (pH 7.2) for 2 h. Following dehydration in a graded series of ethanol, the specimens were transferred to propylene oxide and embedded in Epon (TAAB Laboratories Equipment Ltd., Aldermaston, UK). Ultrathin sections were cut with a Reichert-Jung Ultracut E microtome (Leica Microsystems), collected on 1-hole copper grids with formvar supporting membranes (Merck, Darmstadt, Germany) and stained with uranyl acetate and lead citrate. A Philips CM 100 transmission electron microscope (Philips, Amsterdam, the Netherlands) was used to view the sections, and digital images were obtained with an Olympus Soft Imaging Solutions (OSIS) Veleta side-mounted CCD camera (Olympus, Tokyo, Japan).

### Determining fibre type

From each individual, one cryosection from the control and one from the stimulated leg were analysed (*n* = 3). A total of 70–80 fibres per section were classified according to the presence or absence of MyHCI, MyHCII, MyHCn and MyHCe and the pattern of staining observed at the four ATPase pH levels (ATPase 4.37, ATPase 4.53, ATPase 4.58 and ATPase 10.3).

## Results

### Unambiguous fusion/branching evident in serial cryosections of regenerating muscle

Muscle tissue from healthy individuals who had been subjected to 200 electrically stimulated eccentric (lengthening) contractions 30 days earlier was analysed at the ultrastructural level (TEM), with 3-dimensional reconstructions of single fibre confocal images, and with a thorough study of 22 serial cryosections stained with a variety of immature and mature myosin types as well as ATPase histochemistry.

**Fig. 2:**
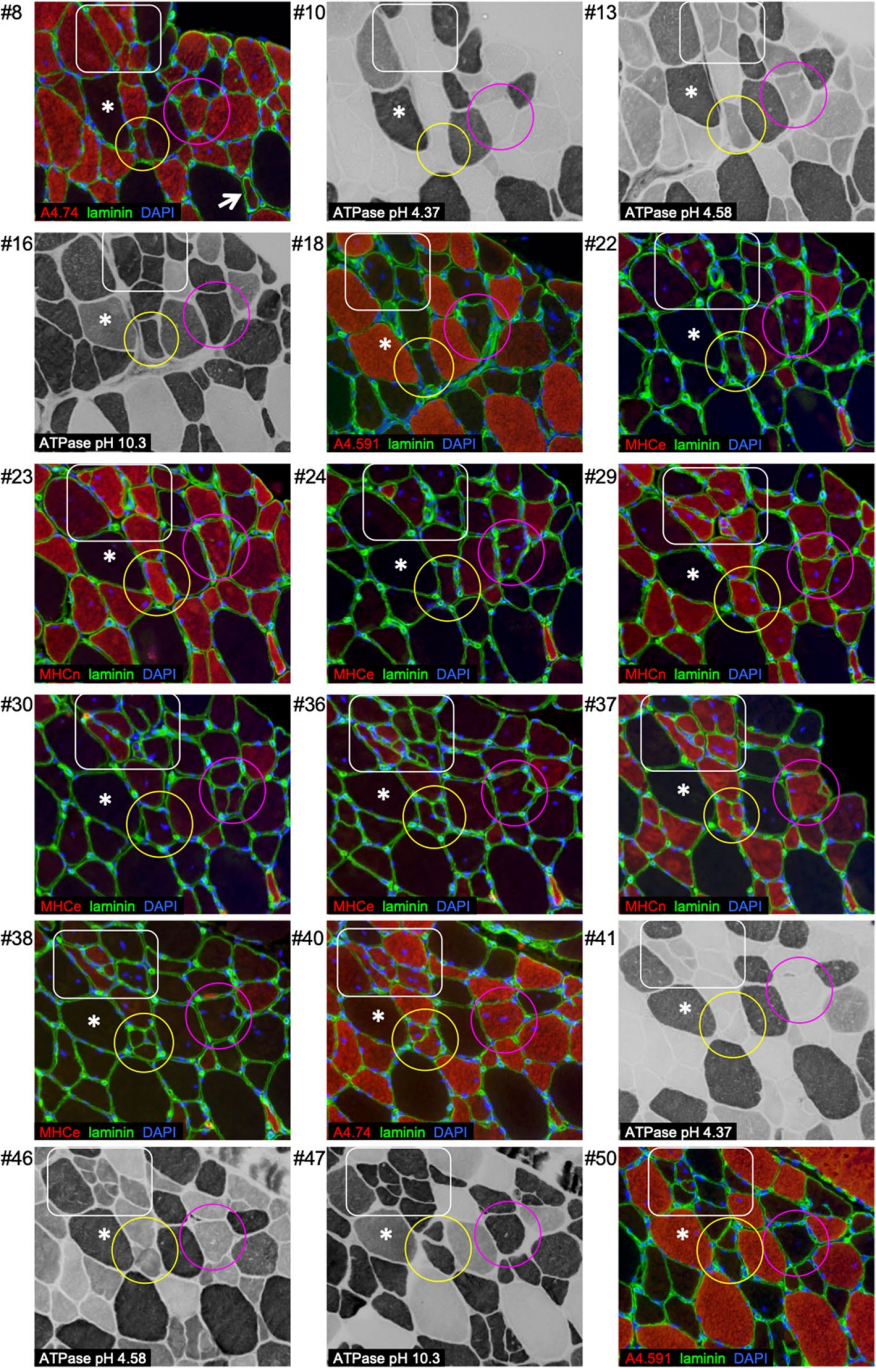
Cross-sectional profiles of regenerating fibres. Serial sections of a biopsy from regenerating human vastus lateralis skeletal muscle 30 days after injury induced by electrical stimulation-eccentric contractions. Sections were stained by ATPase or immunofluorescence, as indicated. In addition to features of branching and fusion, note the high prevalence of fibres positive for MyHCn (and to a lesser extent MyHCe), which mostly appear to have a type II fibre profile (see Fig. 5 for details). Coloured shape outlines highlighted fibres demonstrating branching and/or fusion along this series. *Indicates the same uninjured type I myofibre on each section, for reference. Scale bar, 100 μm

Immunofluorescence images show clear encroachment of the sarcolemma and basement membrane, into some myofibres. Following the same fibres along a series of cryosections, many examples of “branching/splitting” were observed (Figs. 1 and 2). In some cases, these branches reconnected with the parent myofibre at another level in the tissue, and some remained independent. For example, in Fig. 1, the pink circle captures a fibre which from section no. 24 shows the sarcolemma protruding into the fibre (branching point), and from section no. 26, it appears that the fibre has split completely into three fibres. These three fibres remain visible until a further split by section no. 30. On section no. 31, the four fibres are each surrounded by their own sarcolemma. However, on section no. 40, there is only one fibre, approximately equal in size to the sum of the four smaller fibres visible on section no. 31, indicating therefore that the four fibres have fused. On sections no. 40 and no. 50, this fibre exhibits a normal shape and size with respect to the surrounding fibres in this sample.

The additional immunofluorescence stainings provide further information about this fibre and its branches (Fig. 2). They all demonstrate strong immunoreactivity for MyHC II and MyHCn and are negative for MyHC I and MyHCe. The ATPase staining supports the classification of this fibre as type II, which was a common observation in our samples. Of the 210 regenerating fibres that were stained and analysed, only one regenerating fibre predominantly presented as a type I fibre. Similar patterns are evident in the fibres marked with additional outline shapes (Figs. 1 and 2).

In general, branches presented with the same myosin expression profile as their parent myofibre. Occasionally, though, a fibre branch presented as MyHCe + MyHCn + , while the parent myofibre was MyHCe-MyHCn + (Fig. 2, white square). Another consistent observation was that the dystrophin, and laminin staining patterns were similar during branching/fusion, implying a continuous basement membrane with the sarcolemma during this process.

Through the systematic analysis of 12 branching myofibres on cryosections such as those presented in Fig. 2, a total of 21 branches was observed, with a median length of 144 μm (range 24–264 μm). Four branches were still visible 9 sections deeper into the tissue, on the last section collected in our series (section no. 43, Supplemental Table 1). For these 4 branches, we can conclude they have a minimum length of 372μm, with no evidence of refusion with the parent myofibre within this distance.

While fusion/branching events were frequent and unequivocal in our samples, we could not determine from the cryosections whether these events were more likely to be fusion of myotubes or branching/splitting of the main myofibre. To investigate this further, we therefore moved on to more detailed imaging techniques, confocal imaging of single myofibres and TEM.

### The presence of nestin and central myonuclei support fusion rather than branching

**Fig. 3:**
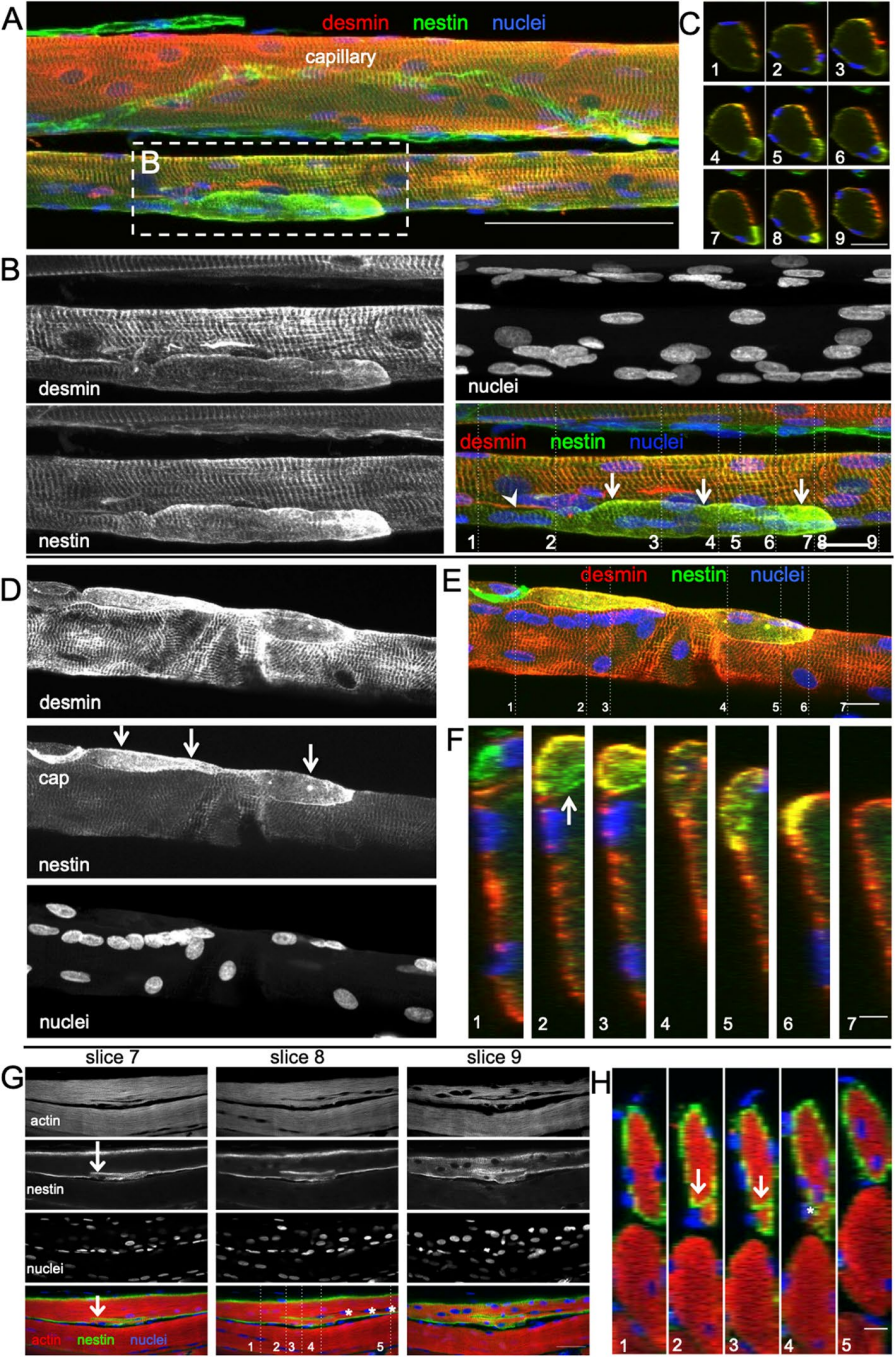
Confocal microscope images of 3 regenerating healthy human muscle fibres, 30 days post injury. **A**–**F** are stained for desmin (red), nestin (green), and nuclei (blue) and **G**–**H** are stained for actin (phalloidin, red), nestin (green) and nuclei (blue). **A** Maximum intensity projection of a 14-slice z-stack (100-μm scale bar), displaying a nestin + branch attached to a regenerating myofibre (the lower myofibre displayed here alongside an (upper) uninjured myofibre). **B** Maximum intensity projection of a 25-slice z-stack (20-μm scale bar). Note the striated and nestin + segment (arrows) tightly associated with the parent myofibre. This branch displays a gradual increase in nestin immunoreactivity from the point of branching (or fusion) towards its end (**C** orthogonal slices 7–8). **D**–**E** Maximum intensity projections of a 10-slice z-stack (20-μm scale bar). Note the small nestin + desmin + myofibre segment (arrows) nestled against the parent myofibre (nestin-desmin +). Note the approximately 10 juxtaposed myonuclei in **D**. **G** Three slices of a z-stack (scale bar, 50 μm), showing 2 myofibres. The presence of nestin at the perimeter of the upper myofibre in this image indicates ongoing regeneration, in contrast to the lower myofibre. Arrows point to a region of the regenerating myofibre that appears to be split for a length of approximately 100 μm, demarcated by nestin (**H**). It can be seen from the striated actin staining that the smaller segment is longitudinally continuous with the parent myofibre. *Central myonuclei potentially indicate the site of recent fusion. The position of the YZ orthogonal view images in **C** (20-μm scale bar), **F** (scale bar 5 μm) and **H** (scale bar 10 μm) is designated by dashed lines in **B**, **E** and **G**, respectively

High-resolution confocal microscopy in 3 dimensions not only confirmed the evidence of branches seen in the cryosections but also provided additional information. Since the fixation procedure used to prepare these fibre bundles is not conducive to MyHCe and MyHCn immunofluorescence, we stained for nestin, a protein only found in regenerating or denervated myofibres, or confined to the NMJ and MTJ in the unperturbed state [32, 33]. As seen in Fig. 3A–F, the myofibre branches display strong cytoplasmic immunoreactivity to nestin, where in particular the first myofibre branch displays most intense nestin signal towards the end of the branch (Fig. 3A–B). Although striations representing sarcomeres are visible in the nestin image, this same branch presents a striking lack of desmin + striations, in contrast to the desmin staining pattern of the parent fibre. Nestin in the branch along with desmin-negative striations suggests the branch is at an earlier stage of regeneration than the parent fibre. Chains of nuclei were common in regenerating single fibres, often coinciding with the point of branching, as reported earlier [30]. This is especially apparent in Fig. 3G–H, where a branch appears to be connected to its parent myofibre at both ends, one of which immediately follows a chain of nuclei in the parent fibre. The sarcomeres of the branch segment in Fig. 3G–H are continuous with the sarcomeres of the fibre proper, and aside from the chain of nuclei, only nestin staining of the sarcolemma of the branch and parent myofibre disclose the incomplete regeneration stage of this fibre. Taken together, branch segments present with an earlier regeneration stage than the regenerating parent myofibre.

### Ultrastructural signs of fusion not branching

**Fig. 4:**
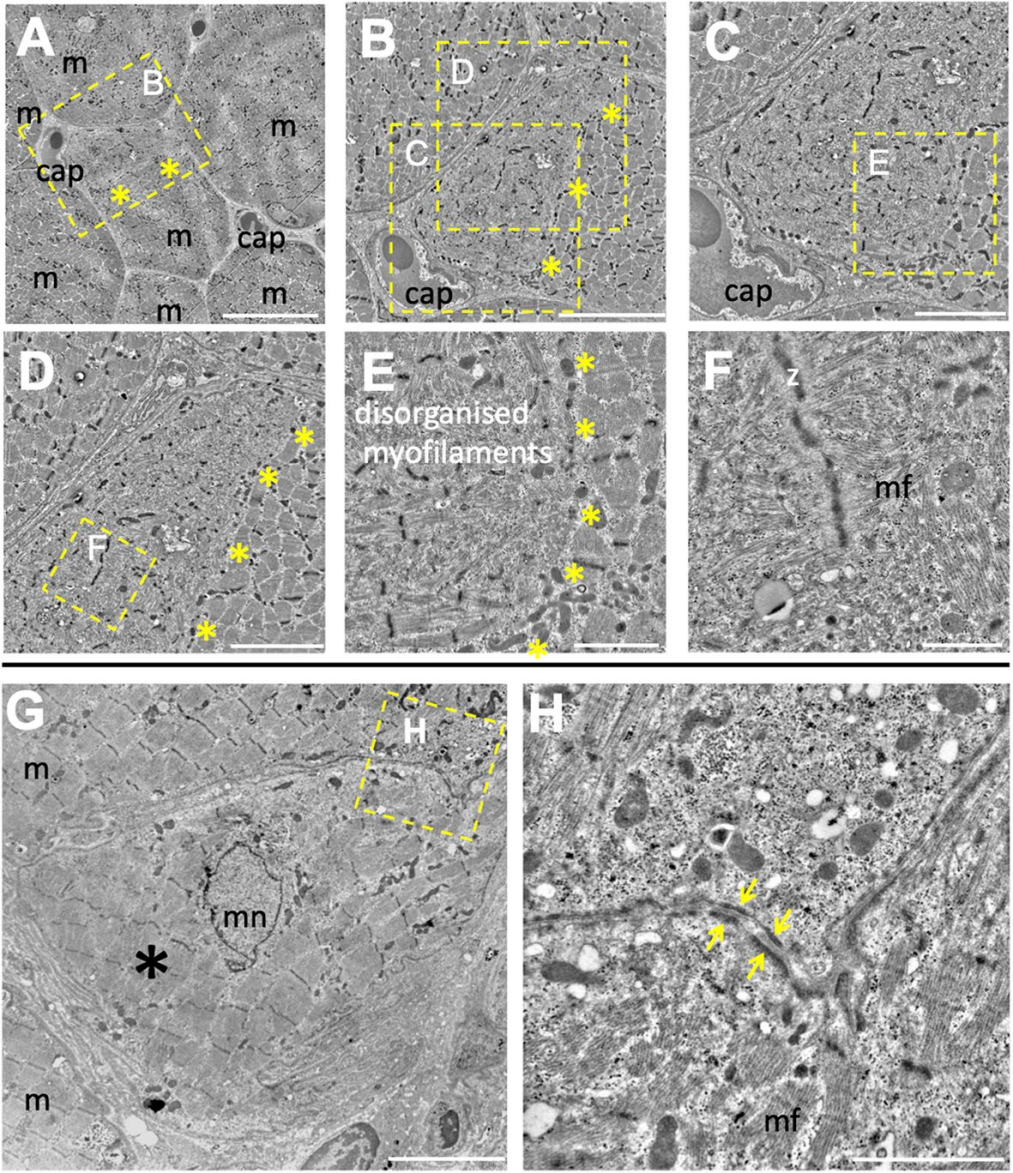
Transmission electron microscopy images of cross-sections of regenerating human muscle, 30 days post injury, in two subjects (one subject **A**–**F**, second subject in **G**–**H**). **A** shows a small fibre surrounded by larger myofibres (20-μm scale bar). **B**–**F** (scale bar 10 μm (**B**), 5 μm (**C**), 5 μm (**D**), 2 μm (**E**), 1 μm (**F**)) show magnified images of **A**. **G** A myofibre branch (*) closely associated with a parent myofibre (scale bar 10 μm), with evidence of membrane fusion, further magnified in H (scale bar 2 μm). The arrows point to membrane-associated electron-dense plaques, indicative of membranes in the phase of fusing. In all images, Z is z-disc, cap is capillary, m is myofibre, mn is myonucleus and mf is myofilament

Cross-sections from two subjects were imaged by transmission electron microscopy (Fig. 4). In Fig. 4A–F, a small myofibre is surrounded by larger myofibres. In the higher magnification images (Fig. 4B–F), it is clear that this smaller myofibre predominantly contains organised myofibrils, but also a relatively large area of disorganised myofilaments, corresponding to approximately one-third of the area of the fibre. The yellow asterisks demarcate the non-membranous border between these two zones. Notably, mitochondria align with this border, potential remnants of a population of sarcolemmal mitochondria from the time when this disorganised zone was a sarcolemma-defined branch, separate from its parent myofibre. Additional evidence of fusion, rather than branching, is presented in the high magnification Fig. 4H in the form of membrane-associated electron-dense plaques, reported in drosophila as necessary for myoblast fusion [34].

### Myosin maturation states of regenerating fibres

**Fig. 5:**
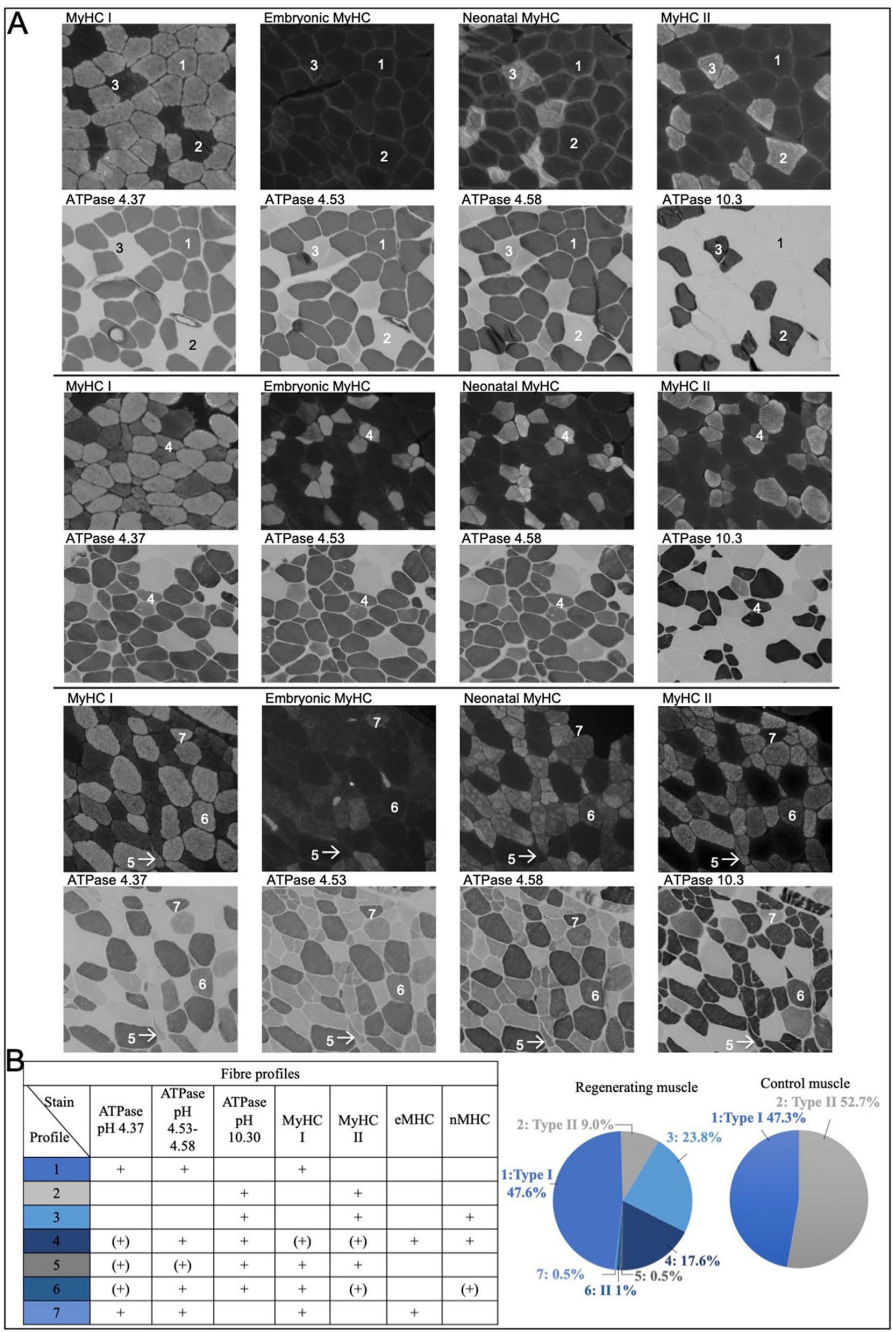
**A** Staining patterns for myosin heavy chain (MyHC) I, II, embryonic (e) and neonatal (n) (stained fibres are light, and unstained fibres are dark) and ATPase staining at pH 4.37, 4.53. 4.58 and 10.3 (stained fibres are dark, and unstained fibres are light). The numbered fibres fall into one of 7 profiles which are quantified in **B**, where the proportions are displayed for regenerating muscle and control muscle

Thirty days post electrical stimulation, while regeneration is well underway, it is clearly incomplete, and there is heterogeneity within a sample regarding stage of regeneration, with regard to the presence of mature (MyHC I and II) and immature (MyHCe, MyHCn) myosin types. We created additional myosin profiles to those observed in rested muscle samples to accommodate all myofibre profiles present in our specimens, based on the staining patterns on serial sections for MyHC I, MyHC II, MyHCe, MyHCn and ATPase staining at pH levels 4.37, 4.53. 4.58 and 10.3 (Fig. 5A). A total of 210 regenerating muscle fibres and 182 control muscle fibres from 3 subjects was analysed. The control fibres fell into the two classic type I or type II fibre classifications, while the regenerating fibres presented with different patterns, requiring a total of 7 staining profiles (Fig. 5A–B). Several findings are worth noting. Firstly, a similar proportion of type I fibres (47%) was detected in samples from the control and stimulated conditions, suggesting type I fibres were not damaged by the stimulation protocol. Secondly, only 9% of fibres in the stimulated leg could be categorised as classic type II fibres, corresponding to 53% in the control leg of these same individuals. The remaining 44% of fibres in the stimulated leg were represented by two major type II fibre profiles, both of which were positive for MyHCe and MyHCn. Of these, one category was also positive for MyHC I, while the other was negative for MyHC I, making up 18% and 24%, respectively, of the total fibre pool. The remaining three categories represent patterns which are only found in 1–2 regenerating fibres each and which could not be assigned to any of the existing fibre groups. These fibres did not reflect a clear type I or II fibre type, with conflicting ATPase and immunoreactivity staining patterns.

## Discussion

The occurrence of hyperplasia in healthy adult skeletal muscle is widely debated, fuelled by observations of “splitting” myofibres. However, in principle, such features could be explained by fusion of a myotube to its “parent” myofibre. In our detailed examination of healthy human muscle undergoing adult regenerative myogenesis after myofibre necrosis, we find support for fusion rather than splitting, which argues against hyperplasia, in line with earlier hypotheses that observations of branching/splitting represent fusion and are a physiological process in healthy muscle.

**Fig. 6:**
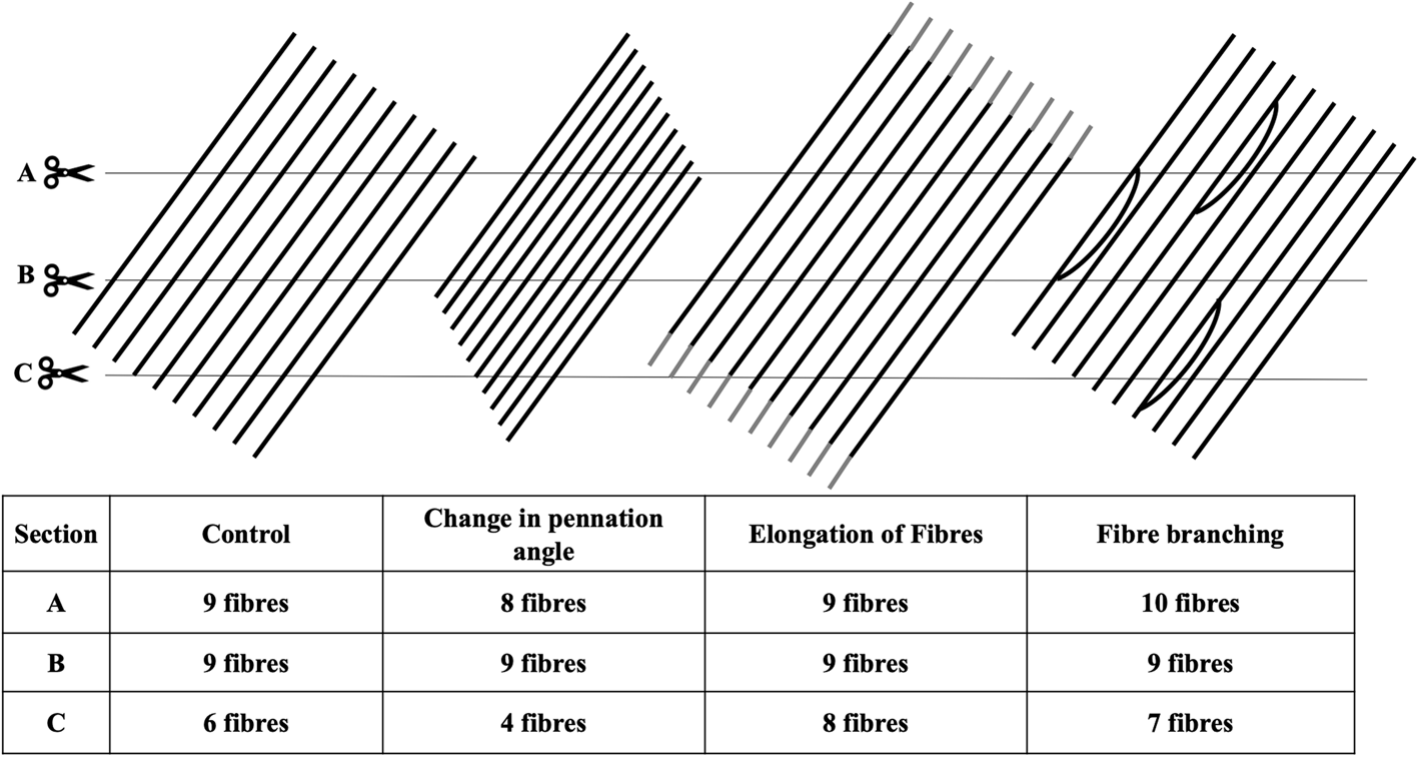
Illustration of the challenges of counting myofibre number on muscle cross-sections, due to changes in pennation angle, myofibre length and myofibre branching

It is important to note that our study examines regenerating myofibres, as shown by positive staining for the developmental myosins (neonatal and embryonic MHC), in healthy adult skeletal muscle. While there are some parallels between embryonic development and adult regenerative myogenesis [35], our findings may not necessarily represent other situations, such as development or muscle growth following heavy loading. This distinction is important, as data in larva show that, specifically during metamorphosis, fibre splitting is a common feature in this species [15]. However, more commonly, changes in muscle mass are investigated following heavy loading, a situation characterised by repair of segmental muscle damage or growth. In any case, changes in muscle mass are most often observed by looking at cross-sections of either whole animal muscles or muscle biopsies in humans. In a comprehensive review from 2019, Murach and colleagues describe fibre splitting following extreme loading conditions in animals [7]. Among others, they use data from Roy and Edgerton [36] to illustrate how changes in fibre pennation angle can make it difficult to assess fibre number from a tissue cross-section. While this is correct, change in fibre length is an additional critical factor, as is pointed out in a letter to the editor by Jorgenson and Hornberger [37]. Fibre lengthening is primarily seen; following eccentric resistance training [38] represents an additional challenge to counting fibres on muscle cross-sections. In this study, we show that branching seen during regeneration can further complicate this analysis. Figure 6 is a schematic representation, inspired by the illustrations by Murach et al. [7] and Jorgenson and Hornberg [37] of how changes to muscle morphology, and the angle and depth of the cross section, can influence total fibre number counts. This emphasises that under non-control situations, e.g. following training, during regeneration or in pathological states, muscle cross sections should be used with caution if the goal is to count the total number of fibres in a muscle. Furthermore, these methodological issues have likely clouded the data pertaining to hyperplasia.

We set out to assess whether muscle fibre branching represents complete fibre splitting, and, with this, myofibre hyperplasia, or alternatively if the presence of branching fibres could be explained by fusion of myotubes rather than splitting. In this systematic analysis of regenerating human muscle, 30 days after injury induced by electrical stimulation, we find multiple signs of fibre splitting or branching, in line with earlier reports in powerlifters and anabolic drug users [19, 20]. By following these fibres through a series of 22 consecutive sections, we frequently observed the branch fusing with the parent myofibre again (Fig. 1), with a median branch length of 144 μm (range 24–264 μm). The same fibre thus appears split in portions of its length and “normal” in other parts. We often observed a single fibre branching into several smaller fibres, similar to observations in rodents in pathological states [4] and following strenuous exercise [10]. This has previously been described as evidence of muscle fibre hyperplasia. Thus, when examined alone, a single cross section can be difficult to interpret.

While the serial sectioning analysis confirmed branching as a regular feature at this time point of 4 weeks post injury, a continuation of this process towards complete splitting could still be a viable explanation for our observations. To investigate further, we turned to high-resolution confocal imaging for viewing single fibres longitudinally in 3 dimensions. Small myofibre branches were observed, tightly associated with parent myofibres. These branches were often positive for neonatal and in some cases embryonic myosin (Fig. 2), as well as staining positive for nestin, which has been shown to stain fibres in the regenerating or un-innervated states [32, 33, 39]. Importantly, both the neonatal myogenic marker and nestin-positive staining indicate that the branch is at an earlier stage of development in the regeneration process than the parent fibre. This is further supported by the lack of desmin + striations in these branch segments (Figs. 3 and 4). Disruption to the sarcomeric striations reported by Crameri and colleagues in the days after maximal eccentric voluntary exercise [40] is a sign of fibre damage and degeneration, while at the 4-week time point in the present study, it represents a different process — the formation of a new myofibre. Theoretically, fibre splitting would result in two, or more, similar fibres, rather than one fibre with mature sarcomeres and one without. Therefore, we find it most likely that the fibres are not splitting and dividing, but rather that the branch is in an earlier state of regeneration than the parent myofibre, where fusion between the branch and parent myofibre is an ongoing process. The chains of myonuclei extending from the point of branch fusion support this, as these nuclei likely mark the point of recent fusion between branch and parent sarcolemmas. Further substantiation for fusion can be found in the transmission electron microscopy images. Firstly, we observed myofibres with different states of sarcomere arrangement separated by non-membranous borders (Fig. 4), again supporting fusion of an immature branch with a more mature parent myofibre. Secondly, between a small myofibre (potentially a branch) and a larger closely associated myofibre, we observed membrane-associated electron-dense plaques. Electron-dense plaques are shown in *Drosophila* to be needed for the initial step of membrane fusion between myoblast and myotube [34, 41, 42]; however, electron-dense plaques are also observed, in kette mutants, where fibres do not fuse [42]. As such, electron-dense plaques are not indicant of fusion but can be interpreted as membranes in the phase of fusing. Taken together, we propose that the branching signs we, and others, observe can be explained by incomplete, ongoing, regeneration following fibre necrosis, and not splitting of myofibres leading to hyperplasia.

How these branches occur in the first place deserves some consideration. The appearance of split or branched myofibres has earlier been attributed to as “incomplete lateral fusion of myotubes during regeneration” [21, 22], which fits well with our understanding of how necrotic muscle fibres are replaced by new myofibres. Successful muscle regeneration requires the preservation of the myofibre basement membrane. The original basement membrane is eventually shed after providing essential scaffolding for myogenesis [16, 43]. Basement membrane formation distinguishes foetal muscle development from adult myogenesis because the basement membrane does not form until later stages of muscle development [43]. Within the original basement membrane, satellite cells are activated, proliferate, differentiate and eventually fuse with each other to form myotubes. Thus, one basement membrane scaffold will contain many myotubes, which in turn fuse with each other to form a single myofibre. With this in mind, it does not seem unreasonable that a myotube occasionally does not fuse in a synchronised manner with the other myotubes and becomes partially orphaned from the parent myofibre, forming its own sarcolemma and basement membrane. Alternatively, it is possible that the branch represents a lone myotube formed at a later stage of regeneration than the other myotubes.

While our observations support incomplete lateral myotube fusion as a possible explanation for the presence of branching myofibres in healthy regenerating muscle, alternative interpretations should be considered. The branches that are followed from the first point of branching (or fusion), through the 22 consecutive sections, do in some cases fuse again with the parent myofibre, while other branches were not observed to refuse and remained as branches with only a single point of attachment to the parent myofibre. This could be explained by the fibres not being followed far enough to detect fusion with the parent myofibre; however, it is also possible that these branches do not fuse, but rather split, for instance via myocyte grafting as suggested by Murach and colleagues [7]. A dual response could be expected if some fibres suffer only partial destruction. However, from a previous analysis in muscle biopsies taken from these same subjects 7 days following injury, we know that all fibre fragments studied were either completely normal or regenerating (alternating necrotic and regenerating zones, with macrophage infiltration) along their entire length [24], so segmental damage does not seem to be a feature of the electrical stimulation model used, in healthy humans, in the present study. Furthermore, based on the earlier regeneration state of the branch, the centralised nuclei potentially marking site of recent fusion between the branch and parent myofibre (Fig. 3), and the presence of electron dense plaques (Fig. 4) pointing towards the cells being on a path towards fusion, we find it more likely that fusion is ongoing rather than splitting. Whether some branches undergo fusion, while others split completely, could certainly be a feature of other models of muscle overload, injury or pathology, and are potentially species specific.

One of the other interesting observations from the present study was the difficulty in categorising regenerating myofibres as clear type I or type II. The fibres in the non-stimulated leg were straightforward to classify as type I or type II, whereas for the regenerating muscles, we had to create additional profiles, seven in total, most of which expressed developmental myosins which has been well documented previously supporting the notion that there is a re-expression of these during regeneration [44]. The different myosin profiles most likely represent different stages of regeneration, as in the case of the branches, developmental myosin representing an immature state as is seen in the prenatal environment [45]. In general, the MyHC I and II antibody staining was in agreement with the ATPase fibre categories. Although some fibres stained positive with both myosin type I and myosin type II antibodies, in other cases, the ATPase staining profile was inconclusive. Thus, a combination of antibody staining and ATPase staining is helpful for defining a fibre as primarily expressing either type I or type II myosin, under regeneration conditions. The other outcome from this analysis was that the regenerating fibres were almost exclusively type II. Crameri and colleagues have previously suggested, but were not able to show conclusively, that electrical stimulation primarily targets type II fibres [46]. However, Gregory and Bickel suggest in a perspectives paper [47] that it is more likely that electrical stimulation has a stochastic/nonselective fibre-type recruitment pattern when used as a model of exercise. We speculate that the finding that 99.5% of the affected fibres being type II in nature is not a feature of the electrical stimulation model per se but rather a combination of [1] the recruitment pattern seen in the case of very high strain; in this case, electrical stimulation in combination with eccentric contractions selectively recruits large motor units, at least initially, and with this the fast type II fibres and [2] this regeneration stage, 4 weeks after the stimulated contractions. In rat soleus, a predominantly slow muscle, regenerating fibres initially express fast myosin transcripts, and then switch to slow myosin, upon innervation [48]. This supports type II myosin as the default myosin heavy chain type, while the fibre is still developing (and not yet innervated). However, it is not known if this occurs in healthy adult human muscle, and it should be noted that the overall percentage of type I fibres (47%) did not differ between the regenerating muscle and the control muscle, which further supports the regenerating fibres being type II in nature. Our observations relating to branching and fusion therefore only reflect events in type II fibres, in a mixed muscle such as the vastus lateralis.

## Conclusions

In this detailed study of branching myofibres in healthy regenerating human skeletal muscle tissue 4 weeks after a necrosis-inducing event targeting type II fibres, we find more evidence for incomplete ongoing myotube fusion rather splitting (and thereby hyperplasia) of myofibres. However, there may be alternative explanations, and further analysis of full single fibres from tip to tip is needed to definitively confirm this.

### Supplementary Information

**Additional file 1: Supplemental Table 1.** Sequence of histochemical and immunofluorescence stainings on 44 serial cryosections. **Supplemental Table 2.** Cryosection antibody combinations. Primary and secondary antibody combinations in the four immunofluorescence staining protocols used on cryosections (outlined in Supplemental table 1). The last column indicates whether fixation was applied before incubation with primary antibodies or after incubation with the secondary antibodies. **Supplemental Table 3.** Single fibre antibodies and dyes. Primary and secondary antibody, and dye, combinations for single fibre immunofluorescence.

## Data Availability

All data will be available upon request.

## Abbreviations

MyHC I: Myosin heavy chain type I
MyHC II: Myosin heavy chain type II
MyHCe: Myosin heavy chain-embryonic
MyHCn: Myosin heavy chain-neonatal
TEM: Transmission electron microscopy

## References

[1] Taylor NA, Wilkinson JG (1986). Exercise-induced skeletal muscle growth. Hypertrophy or hyperplasia?. Sports Med..

[2] Montgomery RD (1962). Growth of human striated muscle. Nature.

[3] Chiakulas JJ, Pauly JE (1965). A study of postnatal growth of skeletal muscle in the rat. Anat Rec.

[4] Isaacs ER, Bradley WG, Henderson G (1973). Longitudinal fibre splitting in muscular dystrophy: a serial cinematographic study. J Neurol Neurosurg Psychiatry.

[5] Faber RM, Hall JK, Chamberlain JS, Banks GB (2014). Myofiber branching rather than myofiber hyperplasia contributes to muscle hypertrophy in mdx mice. Skelet Muscle.

[6] Schmalbruch H (1984). Regenerated muscle fibers in Duchenne muscular dystrophy: a serial section study. Neurology.

[7] Murach KA, Dungan CM, Peterson CA, McCarthy JJ (2019). Muscle fiber splitting is a physiological response to extreme loading in animals. Exerc Sport Sci Rev.

[8] Antonio J, Gonyea WJ (1993). Skeletal muscle fiber hyperplasia. Med Sci Sports Exerc.

[9] Bonilla E, Samitt CE, Miranda AF, Hays AP, Salviati G, DiMauro S, Kunkel LM, Hoffman EP, Rowland LP (1988). Duchenne muscular dystrophy: deficiency of dystrophin at the muscle cell surface. Cell.

[10] van Linge B (1962). The response of muscle to strenuous exercise. An experimental study in the rat. J Bone Joint Surg Br.

[11] Tamaki T, Shiraishi T (1996). Characteristics of compensatory hypertrophied muscle in the rat: II Comparison of histochemical and functional properties. Anatomical Record.

[12] Alway SE, Winchester PK, Davis ME, Gonyea WJ (1989). Regionalized adaptations and muscle fiber proliferation in stretch-induced enlargement. J Appl Physiol.

[13] Sola OM, Christensen DL, Martin AW (1973). Hypertrophy and hyperplasia of adult chicken anterior latissimus dorsi muscles following stretch with and without denervation. Exp Neurol.

[14] Brown LM, Lopez JR, Olsen JA, Rüdel R, Simmons RM, Taylor SR, Wanek LA (1982). Branched skeletal muscle fibers not associated with dysfunction. Muscle Nerve.

[15] Cripps RM, Olson EN (1998). Twist is required for muscle template splitting during adult drosophila myogenesis.

[16] Mackey AL, Kjaer M (2017). The breaking and making of healthy adult human skeletal muscle in vivo. Skelet Muscle.

[17] Eriksson A, Lindström M, Carlsson L, Thornell LE (2006). Hypertrophic muscle fibers with fissures in power-lifters; fiber splitting or defect regeneration?. Histochem Cell Biol.

[18] MacDougall JD, Sale DG, Elder GCB, Sutton JR (1982). Muscle ultrastructural characteristics of elite powerlifters and bodybuilders. Eur J Appl Physiol Occup Physiol.

[19] Kadi F, Eriksson A, Holmner S, Thornell L-E, Kadi F (1999). Effects of anabolic steroids on the muscle cells of strength-trained athletes. Med Sci Sports Exerc.

[20] Eriksson A, Kadi F, Malm C, Thornell LE (2005). Skeletal muscle morphology in power-lifters with and without anabolic steroids. Histochem Cell Biol.

[21] Grounds MD (2014). Therapies for sarcopenia and regeneration of old skeletal muscles: more a case of old tissue architecture than old stem cells. Bioarchitecture Landes Bioscience..

[22] Grounds MD (2014). The need to more precisely define aspects of skeletal muscle regeneration. Int J Biochem Cell Biol.

[23] Mackey AL, Brandstetter S, Schjerling P, Bojsen-Moller J, Qvortrup K, Pedersen MM, Doessing S, Kjaer M, Magnusson SP, Langberg H (2011). Sequenced response of extracellular matrix deadhesion and fibrotic regulators after muscle damage is involved in protection against future injury in human skeletal muscle. FASEB J.

[24] Mackey AL, Kjaer M. The breaking and making of healthy adult human skeletal muscle in vivo. Skelet Muscle. 2017;7. 10.1186/s13395-017-0142-x. 10.1186/s13395-017-0142-x PMC5688812 29115986

[25] Mackey AL, Rasmussen LK, Kadi F, Schjerling P, Helmark IC, Ponsot E, Aagaard P, Durigan JLQ, Kjaer M (2016). Activation of satellite cells and the regeneration of human skeletal muscle are expedited by ingestion of nonsteroidal anti-inflammatory medication. FASEB J.

[26] Bergstrom J (1975). Percutaneous needle biopsy of skeletal muscle in physiological and clinical research. Scand J Clin Lab Invest.

[27] Dahl R, Larsen S, Dohlmann TL, Qvortrup K, Helge JW, Dela F, Prats C (2015). Three-dimensional reconstruction of the human skeletal muscle mitochondrial network as a tool to assess mitochondrial content and structural organization. Acta Physiol.

[28] Brooke MH, Kaiser KK (1970). Muscle fiber types: how many and what kind?. Arch Neurol.

[29] Andersen JL, Aagaard P (2000). Myosin heavy chain IIX overshoot in human skeletal muscle. Muscle Nerve.

[30] Swash M, Schwartz MS (1977). Implications of longitudinal muscle fibre splitting in neurogenic and myopathic disorders. J Neurol Neurosurg Psychiatry.

[31] Ralston E, Lu Z, Ploug T. The organization of the Golgi complex and microtubules in skeletal muscle is fiber type-dependent. http://rsb.info.nih.gov/nih-image/. 10.1523/JNEUROSCI.19-24-10694.1999 PMC6784920 10594053

[32] Vaittinen S, Lukka R, Sahlgren C, Rantanen J, Hurme T, Lendahl U, Eriksson JE, Kalimo H. Specific and innervation-regulated expression of the intermediate filament protein Nestin at neuromuscular and myotendinous junctions in skeletal muscle. Am J Pathol. 1999;154(2):591–600. 10.1016/S0002-9440(10)65304-7. 10.1016/S0002-9440(10)65304-7 PMC1850010 10027416

[33] Vaittinen S, Lukka R, Sahlgren C, Hurme T, Rantanen J, Lendahl U, Eriksson JE, Kalimo H (2001). The expression of intermediate filament protein nestin as related to vimentin and desmin in regenerating skeletal muscle. J Neuropathol Exp Neurol.

[34] Önel SF, Renkawitz-Pohl R (2009). FuRMAS: triggering myoblast fusion in Drosophila. Dev Dyn.

[35] Tajbakhsh S. Skeletal muscle stem cells in developmental versus regenerative myogenesis. J Intern Med. 2009;266(4):372–89. 10.1111/j.1365-2796.2009.02158.x. 10.1111/j.1365-2796.2009.02158.x 19765181

[36] Roy RR, Edgerton VR (1995). Response of mouse plantaris muscle to functional overload: comparison with rat and cat. Comp Biochem Physiol A Physiol.

[37] Jorgenson KW, Hornberger TA (2019). The overlooked role of fiber length in mechanical load-induced growth of skeletal muscle. Exerc Sport Sci Rev.

[38] Franchi MV, Atherton PJ, Maganaris CN, Narici MV (2016). Fascicle length does increase in response to longitudinal resistance training and in a contraction-mode specific manner. Springerplus.

[39] Soendenbroe C, Andersen JL, Mackey AL (2021). Muscle-nerve communication and the molecular assessment of human skeletal muscle denervation with aging. Am J Physiol Cell Physiol.

[40] Crameri RM, Aagaard P, Qvortrup K, Langberg H, Olesen J, Kjær M (2007). Myofibre damage in human skeletal muscle: effects of electrical stimulation versus voluntary contraction. J Physiol.

[41] Dhanyasi N, Segal D, Shimoni E, Shinder V, Shilo BZ, VijayRaghavan K, Schejter ED (2015). Surface apposition and multiple cell contacts promote myoblast fusion in Drosophila flight muscles. J Cell Biol.

[42] Schröter RH, Lier S, Holz A, Bogdan S, Klämbt C, Beck L, Renkawitz-Pohl R (2004). Kette and blown fuse interact genetically during the second fusion step of myogenesis in Drosophila. Development.

[43] Mackey AL, Magnan M, Chazaud B, Kjaer M (2017). Human skeletal muscle fibroblasts stimulate in vitro myogenesis and in vivo muscle regeneration. J Physiol.

[44] Schiaffino S (2018). Muscle fiber type diversity revealed by anti-myosin heavy chain antibodies. FEBS J.

[45] Schiaffino S, Rossi AC, Smerdu V, Leinwand LA, Reggiani C (2015). Developmental myosins: expression patterns and functional significance. Skelet Muscle.

[46] Crameri RM, Aagaard P, Qvortrup K, Langberg H, Olesen J, Kjær M (2007). Myofibre damage in human skeletal muscle: effects of electrical stimulation versus voluntary contraction. J Physiol.

[47] Gregory CM, Bickel CS (2005). Recruitment patterns in human skeletal muscle during electrical stimulation. Phys Ther.

[48] Jerkovic R, Argentini C, Serrano-Sanchez A, Cordonnier C, Schiaffino S (1997). Early myosin switching induced by nerve activity in regenerating slow skeletal muscle. Cell Struct Funct.

