# Supplementary material for: Fusion of myofibre branches is a physiological feature of healthy human skeletal muscle regeneration
Source: Skelet Muscle. 2023 Aug 12;13:13. doi: 10.1186/s13395-023-00322-2 (PMC10422711; doi:10.1186/s13395-023-00322-2)
Supplement: Supplementary file 1 — Additional file 1: Supplemental Table 1. Sequence of histochemical and immunofluorescence stainings on 44 serial cryosections. Supplemental Table 2. Cryosection antibody combinations. Primary and secondary antibody combinations in the four immunofluorescence staining protocols used on cryosections (outlined in Supplemental table 1). The last column indicates whether fixation was applied before incubation with primary antibodies or after incubation with the secondary antibodies. Supplemental Table 3. Single fibre antibodies and dyes. Primary and secondary antibody, and dye, combinations for single fibre immunofluorescence. [file 13395_2023_322_MOESM1_ESM.docx]

**Supplemental tables 1-3.**

Supplemental table 1.

| Slide | Section | Staining | Cumulative distance (μm) |
| --- | --- | --- | --- |
| 1 | 1 | MyHC II | 12 |
|  | 2 | MyHC II | 24 |
| 2 | 3 | ATPase 4.37 | 36 |
|  | 4 | ATPase 4.37 | 48 |
| 3 | 5 | ATPase 4.53 | 60 |
|  | 6 | ATPase 4.53 | 72 |
| 4 | 7 | ATPase 4.58 | 84 |
|  | 8 | ATPase 4.58 | 96 |
| 5 | 9 | ATPase 10.3 | 108 |
|  | 10 | ATPase 10.3 | 120 |
| 6 | 11 | MyHC I | 132 |
|  | 12 | MyHC I | 144 |
| 7 | 13 | MyHCn | 156 |
|  | 14 | MyHCe | 168 |
| 8 | 15 | MyHCn | 180 |
|  | 16 | MyHCe | 192 |
| 9 | 17 | MyHCn | 204 |
|  | 18 | MyHCe | 216 |
| 10 | 19 | MyHCn | 228 |
|  | 20 | MyHCe | 240 |
| 11 | 21 | MyHCn | 252 |
|  | 22 | MyHCe | 264 |
| 12 | 23 | MyHCn | 276 |
|  | 24 | MyHCe | 288 |
| 13 | 25 | MyHCn | 300 |
|  | 26 | MyHCe | 312 |
| 14 | 27 | MyHCn | 324 |
|  | 28 | MyHCe | 336 |
| 15 | 29 | MyHCn | 348 |
|  | 30 | MyHCe | 360 |
| 16 | 31 | MyHCn | 372 |
|  | 32 | MyHCe | 384 |
| 17 | 33 | MyHC II | 396 |
|  | 34 | MyHC II | 408 |
| 18 | 35 | ATPase 4.37 | 420 |
|  | 36 | ATPase 4.37 | 432 |
| 19 | 37 | ATPase 4.53 | 444 |
|  | 38 | ATPase 4.53 | 456 |
| 20 | 39 | ATPase 4.58 | 468 |
|  | 40 | ATPase 4.58 | 480 |
| 21 | 41 | ATPase 10.3 | 492 |
|  | 42 | ATPase 10.3 | 504 |
| 22 | 43 | MyHC I | 516 |
|  | 44 | MyHC I | 528 |

Sequence of histochemical and immunofluorescence stainings on 44 serial cryosections.

Supplemental table 2

|  | Primary antibodies | Secondary antibodies (Alexa Fluor) | Fixation |
| --- | --- | --- | --- |
| MyHC I | MyHC I, Hybridoma Bank, A4.951, mouse IgG1  Dystrophin, Sigma, D8168, mouse IgG2b  Laminin, Dako, Z0097, rabbit | goat anti-mouse IgG2b 488, A-21141  goat anti-mouse IgG1 568, A-21124  goat anti-rabbit 680, A-21076 | prior to primary antibodies |
| MyHC II | MyHC II, Hybridoma Bank, A4.74, mouse IgG1  Dystrophin, Sigma, D8168, mouse IgG2b  Laminin, Dako, Z0097, rabbit | goat anti-mouse IgG2b 488, A-21141  goat anti-mouse IgG1 568, A-21124  goat anti-rabbit 680, A-21076 | prior to primary antibodies |
| MyHCn | MyHCn, Novocastra, NCL-MHCn, mouse IgG1  Dystrophin, Sigma, D8168, mouse IgG2b  Laminin, Dako, Z0097, rabbit | goat anti-mouse IgG2b 488, A-21141  goat anti-mouse IgG1 568, A-21124  goat anti-rabbit 680, A-21076 | after secondary antibodies |
| MyHCe | MyHCe, Hybridoma Bank, F1.652, mouse IgG1  Dystrophin, Sigma, D8168, mouse IgG2b  Laminin, Dako, Z0097, rabbit | goat anti-mouse IgG2b 488, A-21141  goat anti-mouse IgG1 568, A-21124  goat anti-rabbit 680, A-21076 | after secondary antibodies |

Cryosection antibody combinations. Primary and secondary antibody combinations in the four immunofluorescence staining protocols used on cryosections (outlined in supplemental table 1). The last column indicates whether fixation was applied before incubation with primary antibodies or after incubation with the secondary antibodies.

Supplemental table 3

|  | Primary antibodies | Secondary antibodies/dyes (Alexa Fluor) |
| --- | --- | --- |
| desmin/nestin | desmin [Y66], Abcam, ab32362, rabbit  nestin (10c2), Santa Cruz, sc-23927, mouse | goat anti-rabbit 568, A-11036  goat anti-mouse 488, A-11029 |
| actin/nestin | nestin (10c2), Santa Cruz, sc-23927, mouse | goat anti-mouse 488, A-11029  phalloidin 568, A12380 |

Single fibre antibodies and dyes. Primary and secondary antibody, and dye, combinations for single fibre immunofluorescence.
